# Supplementary material for: Cortical neurons exhibit diverse myelination patterns that scale between mouse brain regions and regenerate after demyelination
Source: Nat Commun. 2021 Aug 6;12:4767. doi: 10.1038/s41467-021-25035-2 (PMC8346564; doi:10.1038/s41467-021-25035-2)
Supplement: Supplementary file 2 — Description of Additional Supplementary Files [file 41467_2021_25035_MOESM2_ESM.docx]

**Description of Additional Supplementary Files**

**Title: Supplementary Video 1** – Methodology of axon and myelin tracing.

Description: Flatmounts containing fluorescent subtype-specific axons were immunostained for MBP. Confocal tiled z stacks (675 µm x 675 µm x 40 µm) were acquired at 63x and stitched together. Channels were split and individual axons within the volume were traced (green line) blinded to their myelination status. These axon traces were subsequently loaded into the MBP channel to find myelin sheaths associated with each traced axon.

**Title: Supplementary Video 2** – Dual imaging of PV interneurons and oligodendrocytes.

Description: 3D view of an example imaged region from a *PV-Cre; Ai9; Mobp-EGFP* mouse. Red, PV neurons; cyan, oligodendrocytes.
