# Supplementary material for: Cortical neurons exhibit diverse myelination patterns that scale between mouse brain regions and regenerate after demyelination
Source: Nat Commun. 2021 Aug 6;12:4767. doi: 10.1038/s41467-021-25035-2 (PMC8346564; doi:10.1038/s41467-021-25035-2)

SUPPLEMENTAL FIGURE LEGENDS

Supplemental Figure 1 – Evidence for myelination in neuron specific Cre lines and virally labeled axon populations.

a–j, Coronal hemisections immunolabeled with MBP from brains expressing tdTomato by various Cre lines used in this study and corresponding high-resolution confocal images (< 10 μm maximum z projections) highlighting evidence for myelinated axons. **a–b,** PV-Cre. **c–d,** SOM-Cre. **e–f,** NXPH4-CreER. **g–h,** RBP4-Cre. **i–j,** NTSR1-Cre. Flatmounts and corresponding images of myelinated axons expressing mCherry from AAV injection into the ventromedial (VM, **k–l**), or posterior (PO, **m–n**), nucleus of thalamus. xz or yz slices on the far right correspond to each dotted yellow line in the merged channel images showing optical cross sections of MBP signal surrounding tdTomato- or mCherry-expressing axons. NTSR1 axons were never myelinated (**j**). N = 4 mice per neuron subtype. n = 60 (PV), 62 (VM), 60 (SOM), 61 (PO), 60 (NXPH4), 78 (RBP4), 60 (NTSR1) axons. Scale bars from left to right, 1 mm, 10 μm, 0.5 μm.

Supplemental Figure 1

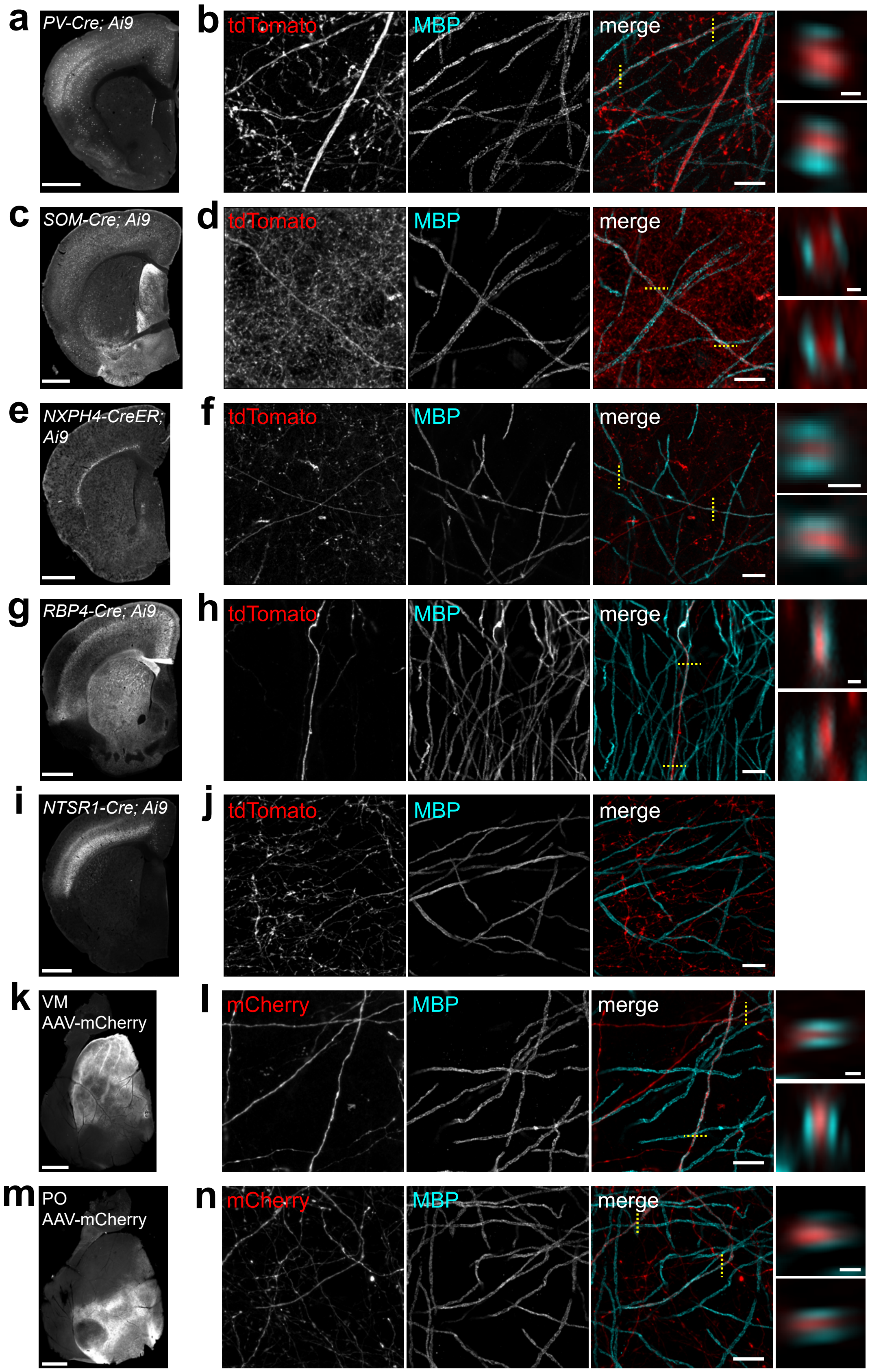

Supplemental Figure 2 – Distributions of cuprizone and control Δ PLM.

a-b, Histograms of the average changes in PLM for PV (**a**) and VM (**b**) axons traced in mice treated with cuprizone (orange, n = 12 regions from 9 mice) and in control mice (blue, n = 5 regions from 3 mice). The control axon distributions are significantly skewed right (few axons showed negative Δ PLM), p = 0.03 (for both PV and VM), two-sample Kolmogorov-Smirnov test. There is no difference in the distributions between cuprizone treated PV and VM axons (p = 0.77 two-sample two-sided Kolmogorov-Smirnov test).

Supplemental Figure 2

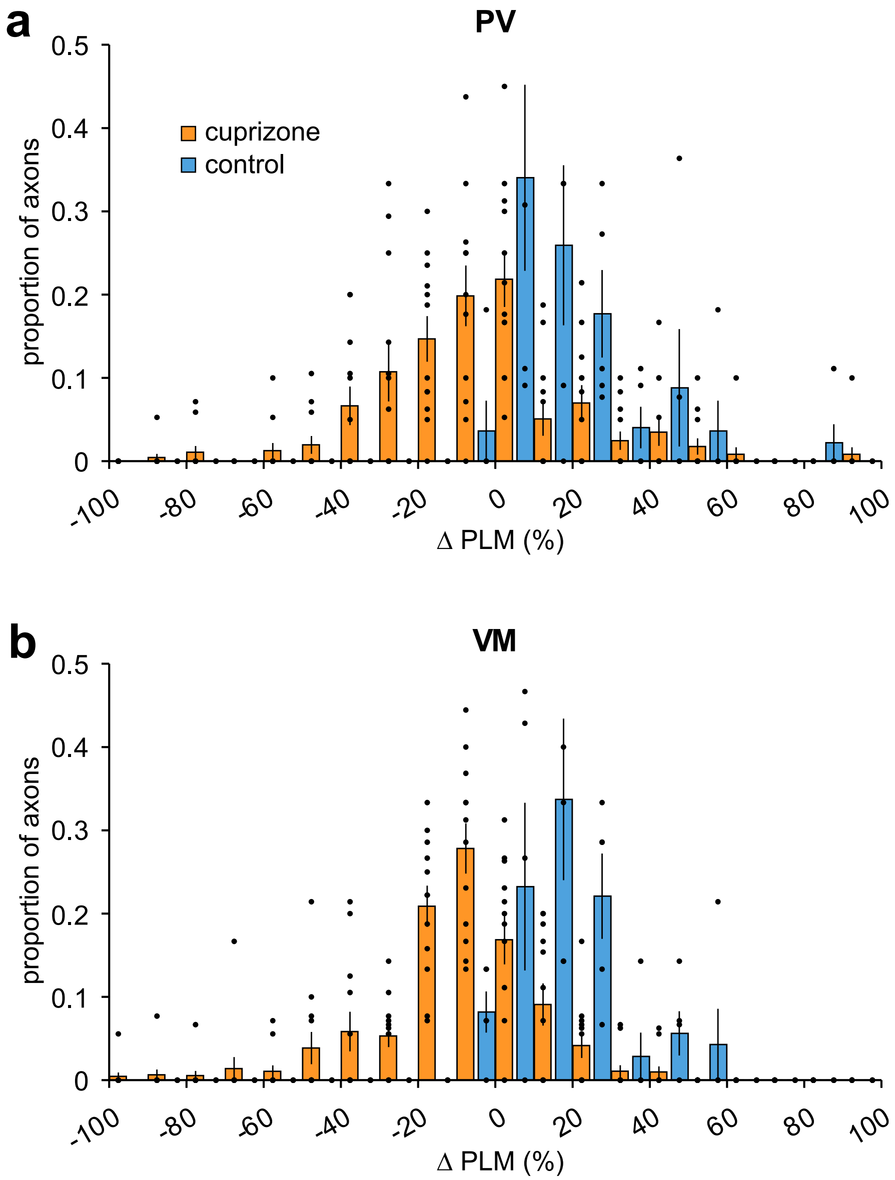

Supplemental Figure 3 – Myelin density is restored by five weeks recovery.

a, Example maximum z projection (55 μm x 55 μm x 15 μm) of SCoRe signal at baseline and five weeks recovery within the same region. **b,** Total number of myelin segments traced between two 100 μm x 100 μm x 30 μm ROIs for each imaged region analyzed in **Figure 5 (**ns, not significant; p = 0.425, paired two-tailed t-test). **c,** Total sum of lengths of all myelin segments for each region in **b** (ns, not significant; p = 0.506, paired two-tailed t-test). n = 2 ROIs each from 12 regions across 9 mice. Scale bar, 10 μm.

Supplemental Figure 3

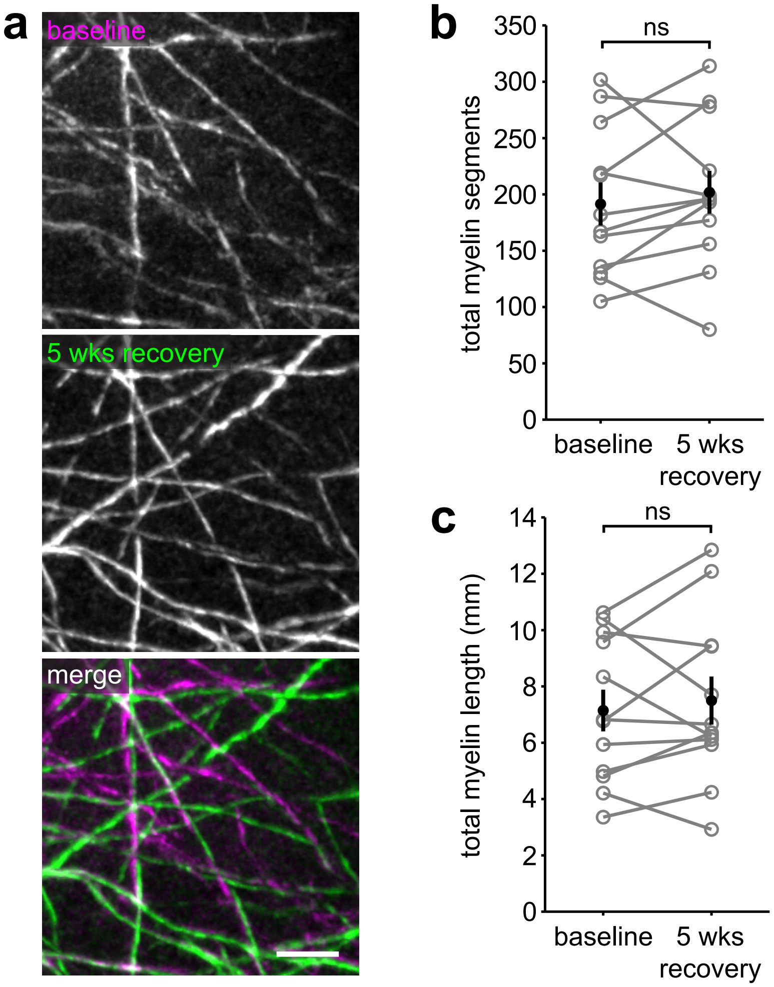

Supplement: Supplementary file 3 — Supplementary Information [file 41467_2021_25035_MOESM3_ESM.pdf]
