# Supplementary material for: Cortical neurons exhibit diverse myelination patterns that scale between mouse brain regions and regenerate after demyelination
Source: Nat Commun. 2021 Aug 6;12:4767. doi: 10.1038/s41467-021-25035-2 (PMC8346564; doi:10.1038/s41467-021-25035-2)
Supplement: Supplementary file 6 — Reporting Summary [file 41467_2021_25035_MOESM6_ESM.pdf]

nature portfolio

Corresponding author(s): Dr. Dwight Bergles

Last updated by author(s): Jun 23, 2021

Reporting Summary

Nature Portfolio wishes to improve the reproducibility of the work that we publish. This form provides structure for consistency and transparency in reporting. For further information on Nature Portfolio policies, see our Editorial Policies and the Editorial Policy Checklist.

Statistics

For all statistical analyses, confirm that the following items are present in the figure legend, table legend, main text, or Methods section.

n/a	Confirmed	
☐	☒	The exact sample size (*n*) for each experimental group/condition, given as a discrete number and unit of measurement
☐	☒	A statement on whether measurements were taken from distinct samples or whether the same sample was measured repeatedly
☐	☒	The statistical test(s) used AND whether they are one- or two-sided<br>*Only common tests should be described solely by name; describe more complex techniques in the Methods section.*
☐	☒	A description of all covariates tested
☐	☒	A description of any assumptions or corrections, such as tests of normality and adjustment for multiple comparisons
☐	☒	A full description of the statistical parameters including central tendency (e.g. means) or other basic estimates (e.g. regression coefficient) AND variation (e.g. standard deviation) or associated estimates of uncertainty (e.g. confidence intervals)
☐	☒	For null hypothesis testing, the test statistic (e.g. *F*, *t*, *r*) with confidence intervals, effect sizes, degrees of freedom and *P* value noted<br>*Give P values as exact values whenever suitable.*
☒	☐	For Bayesian analysis, information on the choice of priors and Markov chain Monte Carlo settings
☒	☐	For hierarchical and complex designs, identification of the appropriate level for tests and full reporting of outcomes
☒	☐	Estimates of effect sizes (e.g. Cohen's *d*, Pearson's *r*), indicating how they were calculated

Our web collection on statistics for biologists contains articles on many of the points above.

Software and code

Policy information about availability of computer code

Data collection	Raw images were collected on ZEISS 710, 800, and 880 microscopes running ZEN Black (2012 SP5 and 2.3 SP1) or ZEN Blue (Zen 2.3). MATLAB (2019b, 2020b) scripts and ImageJ (Fiji, 1.53c) macros are available at https://github.com/clcall/Call_Bergles_2021_CTSM.
Data analysis	Microsoft Excel (365 A5) summary data, MATLAB (2019b, 2020b) scripts, and ImageJ (Fiji, 1.53c) macros are available at https://github.com/clcall/Call_Bergles_2021_CTSM. Volumetric analysis used syGlass v. 1.6.0.

For manuscripts utilizing custom algorithms or software that are central to the research but not yet described in published literature, software must be made available to editors and reviewers. We strongly encourage code deposition in a community repository (e.g. GitHub). See the Nature Portfolio guidelines for submitting code & software for further information.

Data

Policy information about availability of data

All manuscripts must include a data availability statement. This statement should provide the following information, where applicable:

- Accession codes, unique identifiers, or web links for publicly available datasets
- A description of any restrictions on data availability
- For clinical datasets or third party data, please ensure that the statement adheres to our policy

All data will be shared on an unrestricted basis; requests should be directed to the corresponding author. In addition to code, raw tracing files are also available at https://github.com/clcall/Call_Bergles_2021_CTSM.

March 2021

Field-specific reporting

Please select the one below that is the best fit for your research. If you are not sure, read the appropriate sections before making your selection.

☒ Life sciences ☐ Behavioural & social sciences ☐ Ecological, evolutionary & environmental sciences

For a reference copy of the document with all sections, see nature.com/documents/nr-reporting-summary-flat.pdf

Life sciences study design

All studies must disclose on these points even when the disclosure is negative.

Sample size	Traced axon sample sizes were determined by power calculation in a pilot study. Animal sample sizes were chosen based on those reported in previous publications (e.g. Koudelka et al., 2016, Curr Biol; Orthmann-Murphy & Call et al., 2020, eLife).
Data exclusions	No data were excluded.
Replication	All attempts at replication were successful. Four cohorts of mice from different litters were used for in vivo imaging and exhibited similar variance.
Randomization	Time point images were quantified in random order. Axon seeds for tracing were randomly selected within image volumes using randomly generated coordinates in a grid overlaid on the image. Mice used for cuprizone and in vivo imaging experiments were randomly allocated between control and experimental conditions. Sex was distributed evenly between conditions when possible.
Blinding	All experimental analysis was blinded (condition and axon myelination status). Traced morphology was verified by multiple blinded experimenters. Experimenters were not blinded during animal allocation between experimental conditions for in vivo imaging. Instead, mice with suitable windows and fluorescent expression at baseline were evenly distributed between control and experimental conditions.

Reporting for specific materials, systems and methods

We require information from authors about some types of materials, experimental systems and methods used in many studies. Here, indicate whether each material, system or method listed is relevant to your study. If you are not sure if a list item applies to your research, read the appropriate section before selecting a response.

Materials & experimental systems

n/a	Involved in the study	
☐	☒	Antibodies
☒	☐	Eukaryotic cell lines
☒	☐	Palaeontology and archaeology
☐	☒	Animals and other organisms
☒	☐	Human research participants
☒	☐	Clinical data
☒	☐	Dual use research of concern

Methods

n/a	Involved in the study	
☒	☐	ChIP-seq
☒	☐	Flow cytometry
☒	☐	MRI-based neuroimaging

Antibodies

Antibodies used	Chicken anti-GFP Aves Lab GFP-1020 RRID:AB_2307313 1:4000<br>Goat anti-mCherry SicGen AB0040 RRID:AB_2333092; 1:5000<br>Mouse anti-MBP Sternberger 808401 RRID:AB_2564741; 1:2000<br>Chicken anti-MBP Aves Lab F-1005 RRID:AB_2313550; 1:500<br>Anti-Mouse Cy5 Jackson Immuno 715-175-151 RRID:AB_2340820; 1:2000<br>Anti-Goat Cy3 Jackson Immuno 705-166-147 RRID:AB_2340413; 1:2000<br>Anti-Chicken Alexa 488 Jackson Immuno 703-546-155 RRID:AB_2340376; 1:2000<br>Anti-Chicken Cy5 Jackson Immuno 703-006-155 RRID:AB_2340347; 1:2000
Validation	Dilutions were based on those reported by manufacturers and previous published reports from our lab (Hughes & Orthmann-Murphy et al., 2018, Nat Neurosci; Orthmann-Murphy & Call et al., 2020, eLife). Immunostaining results were further validated for each experiment with no-primary controls.

Animals and other organisms

Policy information about studies involving animals; ARRIVE guidelines recommended for reporting animal research

Laboratory animals	Female and male adult mice were used for experiments and randomly assigned to experimental groups. All mice were healthy and did not display any overt behavioral phenotypes. Mice were maintained on a 12-h light/dark cycle, housed in groups no larger than 5, and food and water were provided ad libitum (except during cuprizone-administration, see below). Housing facilities maintained the following conditions: temperature (68°F-79°F); humidity (30%-70% relative humidity). The following transgenic mouse lines were used in this study:<br>Pvalb-IRES-Cre*<br>Sst-IRES-Cre<br>GN220-Ntsr1-Cre<br>Rbp4-KL100<br>Nxph4-2A-CreERT2-D<br>Rosa-CAG-LSL-tdTomato-WPRE (Ai9)*<br>Rosa-CAG-LSL-EYFP-WPRE (Ai3)<br>Mobp-EGFP*<br>*note: C57BL/6 congenic strains were used for cuprizone experiments<br>For axon tracing in immunostained flatmounts, mice were aged to five months. For in vivo imaging experiments mice were 10-12 weeks of age when baseline images were acquired.
Wild animals	This study did not involve wild animals.
Field-collected samples	This study did not use field-collected samples.
Ethics oversight	All animal experiments were performed in strict accordance with protocols approved by the Animal Care and Use Committee at Johns Hopkins University.

Note that full information on the approval of the study protocol must also be provided in the manuscript.
